# Immunoinformatic profiling of parvovirus B19 VP1/VP2 epitopes: relevance to surgical transfusion safety

**DOI:** 10.1097/JS9.0000000000002665

**Published:** 2025-06-05

**Authors:** Maria Karolaynne da Silva, Jonas Galileu Ferreira de Aquino, Emad Rashad Sindi, Md Sarowar Hossain, Magdi E. A. Zaki, João Firmino Rodrigues-Neto, Umberto Laino Fulco, Jonas Ivan Nobre Oliveira

**Affiliations:** a Department of Biophysics and Pharmacology, Bioscience Center, Federal University of Rio Grande do Norte, Natal/RN, Brazil; b Division of Clinical Biochemistry, Department of Basic Medical Sciences, College of Medicine, University of Jeddah, Jeddah, Saudi Arabia; c Computational Biology Research laboratory, Department of Pharmacy, Faculty of Health and Life Sciences, Daffodil International University, Dhaka, Bangladesh; d Chandigarh Pharmacy College, Chandigarh Group of College, Jhanjeri, Mohali, India; e Department of Chemistry, College of Science, Imam Mohammad Ibn Saud Islamic University (IMSIU), Riyadh, Saudi Arabia; f Multicampi School of Medical Sciences, Federal University of Rio Grande do Norte, Caicó/RN, Brazil

**Keywords:** immunodiagnostics, immunoinformatics, parvovirus B19, perioperative care, transfusion safety, viral immunology, VP1 protein, VP2 protein

## Abstract

Human parvovirus B19 (B19V) is a clinically significant pathogen known for its resistance to conventional inactivation methods in blood products, posing a serious risk for transfusion-related transmission particularly in surgical settings and among immunocompromised or pregnant patients. This study employed immunoinformatics tools to identify highly conserved and immunogenic major-histocompatibility-complex (MHC) class I and II epitopes from the VP1 and VP2 capsid proteins, aiming to support the development of targeted immune-based strategies for B19V control. Five MHC class I epitopes and seven class II epitopes were identified, many of which were located within the VP1-unique region, a critical site for viral entry and immune activation. All selected epitopes demonstrated strong antigenicity, non-toxicity, and broad sequence conservation across viral strains, with capacity to elicit robust CD8+ and Th2 responses without inducing IL-10-mediated suppression. These findings provide a foundation for translational research into diagnostic and immune-modulating applications relevant to transfusion safety and perioperative care.

## Introduction

**Figure 1.**
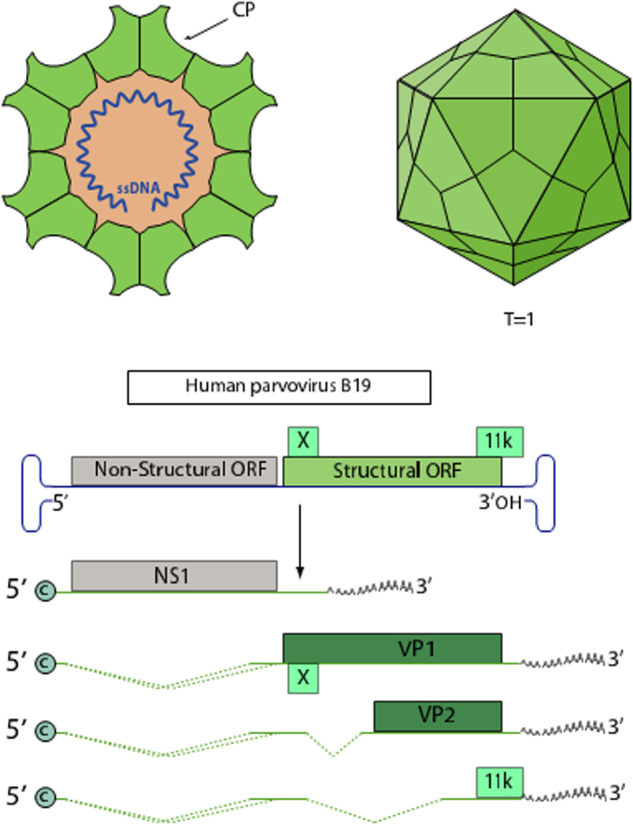
Parvovirus B19 structure and genome. The image shows the icosahedral capsid and ssDNA genome of parvovirus B19, along with its genomic organization and expression of viral proteins NS1, VP1, VP2.

Source: www.viralzone.expasy.org – accessed on April 19, 2025.

Human parvovirus B19 (B19V) is a small, non‑enveloped, single‑stranded DNA virus (*Parvoviridae, Erythroparvovirus*) that encodes NS1, VP1, and VP2 three principal proteins, and shows a strict tropism for erythroid progenitor cells^[1]^. With a genome of approximately 5.6 kb, B19V encodes three main proteins: NS1 (non-structural protein 1), VP1 (viral protein 1), and VP2 (viral protein 2) (Fig. 1)^[1]^. The capsid, composed mainly of VP2 and a smaller fraction of VP1, plays a critical role in viral entry and immune evasion^[1]^. B19V exhibits a strict tropism for erythroid progenitor cells in the bone marrow, leading to various hematological disorders^[2]^. Infection can manifest as erythema infectiosum, transient aplastic crisis, hydrops fetalis and persistent anemia, the latter being particularly problematic for immunocompromised or hematologically fragile patients^[3]^.

**HIGHLIGHTS**

- Human parvovirus B19 remains resistant to standard blood product inactivation, posing a risk in surgical transfusions.
- Using immunoinformatics, five major‑histocompatibility‑complex (MHC)-I and seven MHC-II epitopes from VP1/VP2 were identified as highly conserved and immunogenic.
- Most epitopes are located in the VP1-unique region, a critical domain for viral entry and immune targeting.
- All selected epitopes were non-toxic, non-allergenic, and capable of eliciting robust CD8+ and Th2 responses without IL-10 suppression.
- Findings support the development of epitope-based diagnostics or pathogen-reduction tools to enhance transfusion safety in surgical care.

A critical concern for peri‑operative and critical‑care teams is the inadvertent transmission of B19V through blood transfusions and plasma derivatives. Because of its small, non‑enveloped capsid, B19V resists standard solvent‑detergent and heat‑inactivation protocols^[1]^, creating a residual risk that disproportionately affects surgical, obstetric, and oncological populations who depend on large‑volume or repeated transfusion support. Iatrogenic transmission can prolong hospital stay, precipitate hematological crises, and complicate post‑operative recovery^[4]^.

This clinical challenge is intimately linked to the molecular architecture of the B19V capsid, particularly the structural features of its VP1 and VP2 proteins, which govern both host-cell entry and immune evasion^[1]^. VP1 contains a unique N-terminal region (VP1u) that engages the P-antigen (globoside) receptor and becomes exposed during capsid conformational changes^[5]^. These surface-accessible, highly conserved domains are prime targets for immune recognition, yet natural infection does not always confer lasting protection, and B19V can persist in host tissues for years^[5]^. While NS1 contributes to viral replication, its intracellular location and pro-inflammatory activity make it less attractive for translational applications^[6]^.

Mapping conserved, immunogenic epitopes within VP1/VP2 therefore offers a rational route to several transfusion‑safety solutions: (i) highly specific serological assays to screen donors or recipients, (ii) epitope‑based adsorbents or immunoaffinity filters for pathogen reduction, and (iii) immunotherapeutic or prophylactic formulations for high‑risk surgical cohorts. Leveraging state‑of‑the‑art immunoinformatics, the present study systematically predicts and characterizes major‑histocompatibility‑complex (MHC) class I and II epitopes from VP1 and VP2. By prioritizing epitopes that are conserved, antigenic, non‑toxic and non‑allergenic, we provide a foundation for downstream experimental validation and the development of immune‑guided strategies to mitigate B19V transmission in transfusion‑dependent surgical care.

To enhance the clarity, readability, and academic tone of the manuscript, Instatext was employed during the writing process^[7,8]^. This platform contributed to the linguistic quality of the final text, facilitating more effective communication of the study’s findings, while the core scientific content, analysis, and interpretation remain solely the responsibility of the authors.

## Materials and methods

The VP1/VP2 protein sequences of Parvovirus B19 were retrieved from the National Center for Biotechnology Information^[9]^, specifically from the dataset published in the previous study^[10]^ (accession ID VP1: YP_004928146.1) (accession ID VP2: YP_004928148.1). To mitigate inherent limitations of *in silico* predictions and minimize the risk of false positives, we employed a consensus approach. To identify MHC class I-restricted epitopes, we employed the Immune Epitope Database (IEDB) MHCI binding prediction tool (http://tools.iedb.org/mhci/)^[11]^. This tool predicts epitope binding affinities to human leukocyte antigen (HLA) class I alleles, focusing on those most expressed across diverse populations. For MHC class II epitope identification, we used the IEDB MHCII binding prediction tool (http://tools.iedb.org/mhcii/)^[11]^.

Candidate of MHC-I and MHC-II epitopes were further evaluated for antigenicity using the Vaxijen antigenicity prediction tool (https://www.ddg-pharmfac.net/vaxijen/)^[12]^, ensuring their potential to elicit strong immune responses. For quantitative assessment, a threshold of 0.4 was adopted for antigenicity scores, based on established literature benchmark, and immunogenicity was evaluated by measuring the T-cell activation potential via IEDB. This ensured that only epitopes with robust immune activation were selected. Additionally, the AllerTOP v2.0 server (https://www.ddg-pharmfac.net/allertop_test/)^[13]^ was used to evaluate allergenic potential, while ToxinPred (https://webs.iiitd.edu.in/raghava/toxinpred/index.html)^[14]^ assessed toxicity, which has been widely used in immunoinformatics-based vaccine design against viral pathogens^[15–17]^. Finally, we assessed the ability of the predicted epitopes to induce IL-4 and IL-10 cytokines, which play essential roles in modulating immune responses and immune regulation, using IL4Pred (https://webs.iiitd.edu.in/raghava/il4pred/predict.php)^[18]^ and IL10Pred (https://webs.iiitd.edu.in/raghava/il10pred/predict3.php)^[19]^, respectively, also previously applied in similar contexts^[20]^. Finally, to assess the pro-inflammatory potential of the selected epitopes, we employed the PIP-EL tool (http://211.239.150.230/PIP-EL/)^[21]^, which predicts their ability to induce cytokine release and enhance immune system activation.

Moreover, the IEDB conservancy tool (http://tools.iedb.org/conservancy/)^[11]^ was used to determine sequence conservation across different B19V strains, ensuring broad efficacy. Only epitopes demonstrating 100% conservation across all analyzed B19V strains were retained, thereby ensuring broad applicability and robustness against viral variability. Only epitopes meeting the threshold criteria across multiple platforms were selected. In cases of divergent results, only those epitopes showing consistent outputs across at least two independent methods were prioritized. Future *in vitro* and *in vivo* validations are planned to further substantiate these findings.

## Results and discussion

Despite substantial progress in pathogen inactivation protocols, B19V still poses a meaningful risk of transfusion-transmitted infection, particularly in surgical patients and individuals with weakened immune systems^[22]^. Clinical evidence shows that B19V can evade current filtration methods and nucleic acid testing, especially when donors are asymptomatic carriers^[23]^. As a result, unintended (iatrogenic) transmission continues to occur in high-risk clinical settings^[24]^.

**Figure 2.**
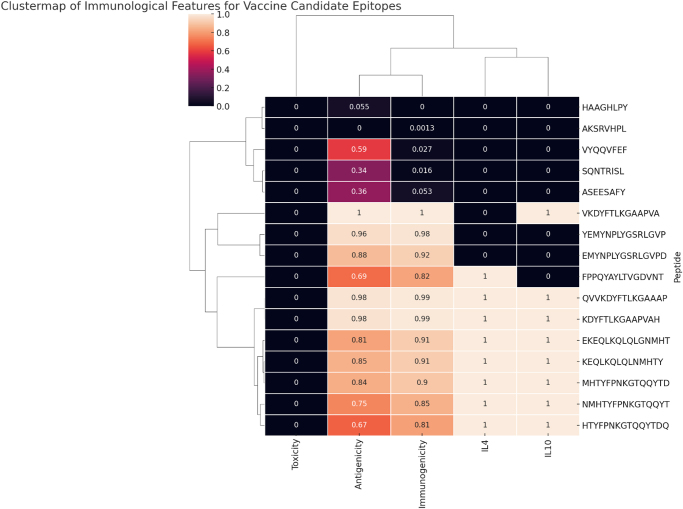
Clustermap showing the classification of all epitopes based on toxicity, antigenicity, immunogenicity, and IL-4/IL-10 induction potential.

To address these limitations, we employed computational profiling of VP1 and VP2 proteins aimed to mitigate these risks through rational epitope identification (Fig. 2). The VP1u, despite comprising only approximately 5% of the capsid, exhibited a disproportionately high immunogenic potential^[5]^. Despite VP2 representing the majority of the capsid (approximately 95%), VP1u has been shown to harbor the most immunodominant and neutralizing epitopes^[5]^. Structural studies suggest that although VP1u is initially internal in the native virion, it becomes exposed under acidic conditions or during cell entry, contributing to its immunogenic profile^[5]^. Individuals lacking strong VP1u-specific responses often exhibit persistent viremia, indicating its essential role in viral clearance^[25]^.

Therefore, incorporating VP1, especially the highly immunogenic regions of VP1u, is crucial for mimicking natural immunity and achieving robust neutralization^[5]^. Interestingly, while this structural and antigenic stability poses it ensures broad cross-strain coverage, it may limit immunological diversity and long-term memory generation, particularly if immune responses are conformationally biased and fail to recognize linear fragments presented in synthetic formulations. This underscores the necessity of preserving native-like epitope configurations in any diagnostic application.

The broad HLA coverage (A and B alleles) increases their potential efficacy across diverse populations, reducing immune escape risks. Given the variability of HLA alleles in different populations, it is important to ensure broad global applicability of the identified epitopes. A recent study analyzing the frequency of HLA classes I and II in 200 populations worldwide revealed considerable genetic diversity, emphasizing the need for population-wide epitope validation^[26]^.

The MHC class I analysis of the VP1/VP2 protein identified four highly conserved epitopes, namely ASEESAFY (HLA-A01:01, antigenicity 0.634), SQNTRISL (HLA-B08:01, antigenicity 0.618), HAAGHLPY (HLA-B35:01, antigenicity 0.461), and AKSRVHPL (HLA-B08:01, antigenicity 0.430) (Table 1). These epitopes demonstrated full or near-full conservation across 86 viral sequences. Like the VP1/VP2 epitopes, they were non-toxic, non-allergenic, and predicted to trigger strong pro-inflammatory responses essential for the induction of durable, protective CD8+ T-cell immunity.

**Table 1:** MHC class I epitope analysis of parvovirus B19 virion proteins

Virion protein 1/2										
Allele	Start	End	Length	Peptide	Antigenicity	Allergenicity	Immunogenicity	Toxicity	Conservancy	PIP
HLA-B*35:01	740	747	8	HAAGHLPY	0.461402	Non-allergen	0.0585	Non-toxin	100%	PIP
HLA-B*08:01	774	781	8	AKSRVHPL	0.430308	Non-allergen	0.05967	Non-toxin	100%	PIP
HLA-A*01:01	432	439	8	ASEESAFY	0.634668	Non-allergen	0.10778	Non-toxin	100%	PIP
HLA-A*24:02	19	26	8	VYQQFVEF	0.760034	Non-allergen	0.0832	Non-toxin	94.19%	PIP
HLA-B*08:01	548	555	8	SQNTRISL	0.61898	Non-allergen	0.07302	Non-toxin	97.67%	PIP

Similarly, the MHC class II epitope analysis of the VP1/VP2 protein identified highly antigenic and fully conserved epitopes (Table 2). Notably, VKDYFTLKGAAAPVA (HLA-DRB101:01, antigenicity 0.9912) and QVVKDYFTLKGAAAP (HLA-DRB101:01, antigenicity 0.9792) emerged as exclusive candidates located within the VP1u, displaying exceptional antigenicity and high sequence conservation. Additionally, epitopes YEMYNPLYGSRLGVP, EMYNPLYGSRLGVPD, KEQLKQLQGLNMHTY, MHTYFPNKGTQQYTD, EKEQLKQLQGLNMHT, NMHTYFPNKGTQQYT, FPPQYAYLTVGDVNT, and HTYFPNKGTQQYTDQ were identified in VP1/VP2 protein, indicating that these regions are highly conserved and structurally accessible across both major capsid components.

**Table 2:** Predicted MHC class II epitopes from parvovirus B19 virion proteins

Virion protein 1/2									
Alleles	Start	End	Peptide	Antigenicity	Allergenicity	Toxicity	Conservancy	IL-4	IL-10
HLA-DRB1*01:01, HLA-DRB1*01:07, HLA-DRB1*01:11, HLA-DRB1*01:13, HLA-DRB1*01:15, HLA-DRB1*01:03	193	207	VKDYFTLKGAAAPVA	0.9912	Non-allergen	Non-Toxin	98.84%	Non-IL4 inducer	IL10 non-inducer
HLA-DRB1*01:01, HLA-DRB1*01:07, HLA-DRB1*01:11, HLA-DRB1*01:15, HLA-DRB1*01:03	194	208	KDYFTLKGAAAPVAH	0.9772	Non-allergen	Non-Toxin	98.84%	Non-IL4 inducer	IL10 non-inducer
HLA-DRB1*01:01, HLA-DRB1*01:07, HLA-DRB1*01:11	477	491	YEMYNPLYGSRLGVP	0.9699	Non-allergen	Non-Toxin	98.84%	Non-IL4 inducer	IL10 non-inducer
HLA-DRB1*01:01, HLA-DRB1*01:07	478	492	EMYNPLYGSRLGVPD	0.9213	Non-allergen	Non-Toxin	98.84%	Non-IL4 inducer	IL10 non-inducer
HLA-DRB1*01:01, HLA-DRB1*01:07	407	421	FPPQYAYLTVGDVNT	0.8163	Non-allergen	Non-Toxin	100.00%	IL4 inducer	IL10 non-inducer
HLA-DRB1*01:01, HLA-DRB1*01:07, HLA-DRB1*01:11, HLA-DRB1*01:13, HLA-DRB1*01:15, HLA-DRB1*01:03	191	205	QVVKDYFTLKGAAAP	0.9792	Non-allergen	Non-Toxin	98.84%	IL4 inducer	IL10 non-inducer
HLA-DRB1*01:01, HLA-DRB1*01:07	605	619	KEQLKQLQGLNMHTY	0.9069	Non-allergen	Non-Toxin	100.00%	IL4 inducer	IL10 inducer
HLA-DRB3*02:02	616	630	MHTYFPNKGTQQYTD	0.9033	Non-allergen	Non-Toxin	100.00%	IL4 inducer	IL10 inducer
HLA-DRB1*01:01, HLA-DRB1*01:07	604	618	EKEQLKQLQGLNMHT	0.8874	Non-allergen	Non-Toxin	100.00%	IL4 inducer	IL10 inducer
HLA-DRB3*02:02	615	629	NMHTYFPNKGTQQYT	0.8505	Non-allergen	Non-Toxin	100.00%	IL4 inducer	IL10 inducer
HLA-DRB3*02:02	617	631	HTYFPNKGTQQYTDQ	0.8048	Non-allergen	Non-Toxin	100.00%	IL4 inducer	IL10 inducer

All selected VP1/VP2 epitopes were non-toxic and non-allergenic, namely QVVKDYFTLKGAAAP, KEQLKQLQGLNMHTY, MHTYFPNKGTQQYTD, and NMHTYFPNKGTQQYT, exhibited IL-4 induction capacity, favoring a Th2 immune response essential for antibody production. None of the identified VP1/VP2 epitopes induced IL-10, suggesting a minimized risk of immune regulation and suppression. The broad HLA-DRB1 and DRB3 allele coverage among these epitopes enhances their immunogenic potential across diverse populations.

The ability of several VP1/VP2 epitopes, such as KEQLKQLQGLNMHTY, MHTYFPNKGTQQYTD, and HTYFPNKGTQQYTDQ, to induce IL-4 suggests their capability to stimulate a Th2-skewed immune response, promoting antibody-mediated viral neutralization. However, the IL-10 induction observed in some shared epitopes highlights the need for further functional studies to fully understand their role in modulating immune activation. Importantly, all VP1/VP2 epitopes were also classified as non-toxic and non-allergenic, supporting their applicability in safe immune-monitoring and intervention strategies.

The identified VP1/VP2 epitopes play a critical role in triggering adaptive immune responses, particularly through the activation of both B and T lymphocytes. Studies have shown that VP1, especially its unique region (VP1u), is highly immunogenic, leading to strong neutralizing antibody responses when presented in virus-like particles^[5]^. Additionally, specific VP1 epitopes, such as 155-FRYSQLAKL-163, have demonstrated high affinity for MHC-I and MHC-II alleles, further enhancing T-cell activation and antibody production^[27]^. These findings emphasize the dual role of these epitopes in generating a robust and durable immune response.

The identified VP1/VP2 epitopes could serve as valuable tools for diagnostic and immunotherapeutic applications. VLPs harboring these epitopes have been proposed as highly sensitive platforms for serological tests to detect B19V-specific antibodies^[28]^. In addition, epitope-based immunotherapies could be tailored to enhance immune responses in immunocompromised individuals or as a targeted strategy for the administration of erythroid-specific drugs^[5]^. These alternative applications emphasize the versatility and importance of these conserved epitopes in viral immunology.

Several translational studies have demonstrated that VP1/VP2 VLPs can induce strong binding and neutralizing antibody responses in preclinical and clinical settings. For example, recombinant VLPs derived from *Saccharomyces cerevisiae* expressing VP1/VP2 elicited potent immune responses in mice, including in models of sickle cell disease^[1]^. Human trials, such as the Phase I and IIa studies by Bernstein *et al*^[29]^, confirmed the safety and immunogenicity of VP1/VP2 formulations. However, cutaneous adverse reactions led to design refinements, including phospholipase A2 inactivation in the VP1u region. These modified VLPs preserved immunogenicity while reducing reactogenicity^[5]^.

Although our epitope selection was grounded in robust immunoinformatics and validated prediction pipelines, real-world translational impact demands validation. *In silico* approaches streamline candidate identification but cannot wholly predict antigen processing, conformational stability, or immunodominance hierarchy *in vivo*. Rapid application of B19V epitope knowledge to transfusion settings remains limited. While point-of-care assays or epitope-coated immunoaffinity filters are technically feasible, they are not yet validated or deployed clinically. Nonetheless, these findings inform the development of advanced diagnostics or pathogen-reduction technologies, particularly in perioperative settings where B19V exposure has critical implications^[30]^.

## Conclusion

This study leveraged immunoinformatics to pinpoint five highly conserved MHC‑I and seven MHC‑II epitopes within the VP1/VP2 capsid proteins of B19V, most of which reside in the VP1‑unique region. All selected epitopes exhibit strong antigenicity, >90% sequence conservation, and no predicted toxicity or allergenicity, indicating their ability to elicit robust CD8+ and Th-skewed responses without IL‑10 mediated suppression. These features position the epitopes as promising molecular targets for immune‑based diagnostics, pathogen-reduction technologies, and other translational strategies aimed at preventing transfusion-related B19V transmission, an issue of particular relevance in surgical and immunocompromised populations.

Validation *in vitro* and *in vivo* remains essential to confirm HLA binding, T-cell activation, and cytokine profiles and to refine epitope selection. Parallel exploration of delivery platforms, such as peptide conjugates, self-amplifying mRNA constructs, or virus-like particle displays, may further optimize immunogenicity and durability. Bridging these computational insights with experimental data will accelerate their translation into clinically useful tools, ultimately enhancing the safety of blood products and perioperative care.

## Data Availability

All essential data were included in the article and supplementary file.
